# Experimental Observation of Long-Range Magnetic Order in Icosahedral Quasicrystals

**DOI:** 10.1021/jacs.1c09954

**Published:** 2021-11-17

**Authors:** Ryuji Tamura, Asuka Ishikawa, Shintaro Suzuki, Takahiro Kotajima, Yujiro Tanaka, Takehito Seki, Naoya Shibata, Tsunetomo Yamada, Takenori Fujii, Chin-Wei Wang, Maxim Avdeev, Kazuhiro Nawa, Daisuke Okuyama, Taku J. Sato

**Affiliations:** † Department of Materials Science and Technology, Tokyo University of Science, Katsushika, Tokyo 125-8585, Japan; ‡ Research Institute for Science and Technology, Tokyo University of Science, Katsushika, Tokyo 125-8585, Japan; § Institute of Engineering Innovation, School of Engineering, The University of Tokyo, Bunkyo, Tokyo 113-8656, Japan; ∥ Department of Applied Physics, Tokyo University of Science, Katsushika, Tokyo 125-8585, Japan; ⊥ Cryogenic Research Center, The University of Tokyo, Bunkyo, Tokyo 113-0032, Japan; # National Synchrotron Radiation Research Center, Hsinchu 30076, Taiwan; % Australian Nuclear Science and Technology Organisation, New Illawarra Road, Lucas Heights, NSW 2234, Australia; & School of Chemistry, The University of Sydney, Sydney, NSW 2006, Australia; @ Institute of Multidisciplinary Research for Advanced Materials, Tohoku University, 2-1-1 Katahira, Aoba, Sendai 980-8577, Japan

## Abstract

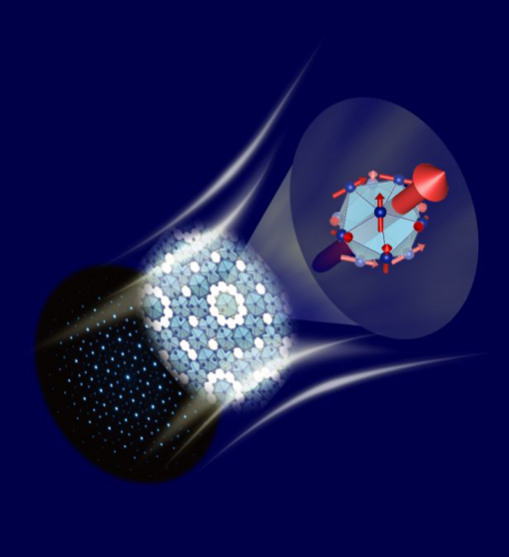

Quasicrystals (QCs), first discovered in 1984, generally do not exhibit long-range magnetic order. Here, we report on long-range magnetic order in the real icosahedral quasicrystals (*i* QCs) Au–Ga–Gd and Au–Ga–Tb. The Au_65_Ga_20_Gd_15_*i* QC exhibits a ferromagnetic transition at *T*_C_ = 23 K, manifested as a sharp anomaly in both magnetic susceptibility and specific heat measurements, along with an appearance of magnetic Bragg peak below *T*_C_. This is the first observation of long-range magnetic order in a real quasicrystal, in contrast to the spin-glass-like behaviors observed for the other magnetic quasicrystals found to date. Moreover, when Gd is replaced by Tb, i.e., for the Au_65_Ga_20_Tb_15_*i* QC, a ferromagnetic behavior is still retained with *T*_C_ = 16 K. Although the sharp anomaly in the specific heat observed for the Au_65_Ga_20_Gd_15_*i* QC becomes broadened upon Tb substitution, neutron diffraction experiments clearly show marked development of magnetic Bragg peaks just below *T*_C_, indicating long-range magnetic order for the Au_65_Ga_20_Tb_15_*i* QC also. Our findings can contribute to the further investigation of exotic magnetic orders formed on real quasiperiodic lattices with *unprecedented* highest global symmetry, i.e., icosahedral symmetry.

## Introduction

**Figure 1:**
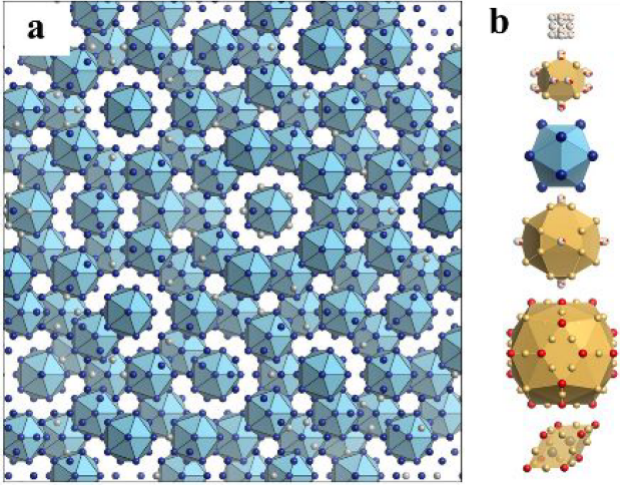
Atomic structure of the Tsai-type icosahedral quasicrystal. (a) Arrangement of the rare-earth (*R*) atoms viewed along a 5-fold axis. The *R* atoms in blue (84.57% of the total *R* atoms) are located at the vertices of the icosahedra whereas those in silver (15.43%) are situated inside the acute rhombohedra (shown at the bottom of part b). (b) Five successive concentric clusters that form the rhombic triacontahedral (RTH) cluster (top) and acute rhombohedron (bottom). The atoms in blue and silver represent the same *R* atoms shown in (a) whereas those in gold and red denote Au and Ga atoms, respectively. The cluster structure is illustrated based on the structure model of the Cd–Yb QC^31^ and Au–Ga–Yb 1/1 approximant,^32^ and the acute rhombohedron is drawn based on the structure model of the Cd–Ca 2/1 approximant.^33^ This image was obtained by using the VESTA 3 program package.^34^

Quasicrystals (QCs) are solids that possess long-range positional order with crystallographically forbidden symmetries such as 5-fold, 10-fold, and 12-fold rotational symmetries (Figure 1). Since the discovery of the Al_86_Mn_14_ icosahedral quasicrystal (*i* QC) in 1984,^1^ researchers have evinced tremendous interest in the physical properties of this new class of ordered solids. However, no physical property directly reflecting the long-range quasiperiodic order has been reported to date; in particular, no long-range magnetic order has been observed thus far. Meanwhile, all magnetic-moment-bearing QCs exhibit a spin-glass-like freezing behavior without exception.^2−17^ In the search for long-range magnetic order in *i* QCs during the past quarter century, researchers have particularly focused on rare-earth (*R*)-containing *i* QCs having well-localized magnetic moments, such as Zn–Mg–*R*,^5−12^ Cd–Mg–*R*,^13−15^ and Cd–*R i* QCs.^16,17^ However, their magnetic susceptibilities commonly display spin-freezing phenomena that are characterized by bifurcation in the zero-field-cooled (ZFC) and field-cooled (FC) susceptibilities without any accompanying sharp anomaly in the specific heat. Here, it is noteworthy that not only antiferromagnetism but also any other long-range magnetic orders such as ferro- and ferrimagnetism have not been observed in real QCs.

The situation had been similar for approximant crystals (ACs), whose atomic configurations closely approximate the local atomic structures in QCs, until a nearly decade ago when antiferromagnetic transitions were discovered in Cd_6_Tb 1/1 AC^18^ and a family of Cd_6_*R* 1/1 ACs.^19^ The observations of antiferromagnetic transitions and also the subsequent finding of ferromagnetic transitions in Au–Si–*R* (*R* = Gd, Tb, Dy, Ho) 1/1 ACs^20,21^ have motivated us to search for magnetically ordered *i* QCs that are likely to exist in the vicinity of the magnetic ACs. Here, to the best of our knowledge, we report on the *first* long-range magnetic order in QCs, obtained by tuning the average electron-per-atom ratio (*e*/*a* = 1.70) near which the strongest ferromagnetism (highest Weiss temperature Θ) was recently observed for Au–Al–Gd 1/1 AC.^22^

## Results

### Search for First Ferromagnetic Quasicrystals

**Figure 2:**
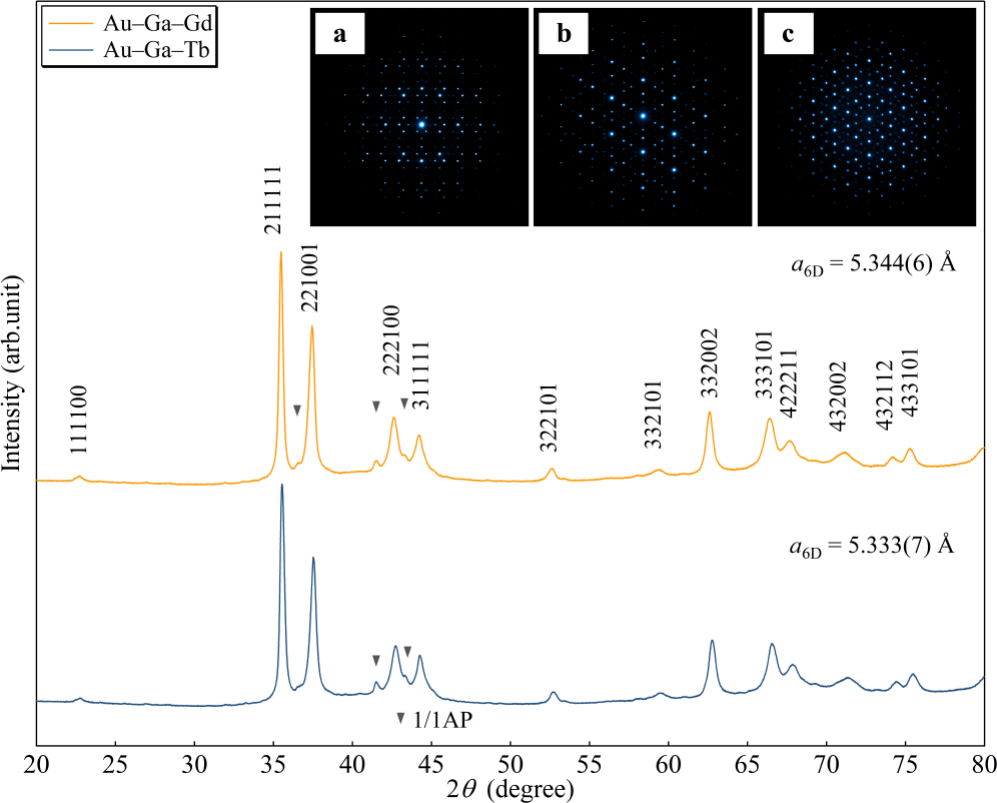
Powder X-ray diffraction patterns for Au_65_Ga_20_Gd_15_ and Au_65_Ga_20_Tb_15_ samples. Mother alloys of various compositions near *e*/*a* = 1.70, prepared by arc-melting, were subjected to rapid quenching onto a Cu wheel rotating at 4000 rpm. As shown in the patterns, icosahedral quasicrystals (*i* QCs) are formed for Au_65_Ga_20_*R*_15_ (*R* = Gd, Tb) compositions with *e*/*a* = 1.70. Most of the peaks are indexed as those of a primitive *i* QC, indicating that a nearly single-phase *i* QC is formed for both samples. The peaks denoted by the triangles are from the 1/1 Au–Ga–*R* approximant. The inset displays selected-area electron diffraction patterns of Au_65_Ga_20_Gd_15_ along the (a) 2-fold, (b) 3-fold, and (c) 5-fold axes, depicting the formation of an *i* QC.

In this study, Au–Ga–*R* alloys prepared with various compositions around *e*/*a* = 1.70 were rapidly quenched to search for new ferromagnetic *i* QCs. Figure 2 presents the powder X-ray diffraction (XRD) patterns of the Au_65_Ga_20_*R*_15_ (*R* = Gd, Tb) samples with *e*/*a* = 1.70; most of the peaks can be identified as *i* QC peaks for both compounds.

For both systems, some peaks are assigned to those of the 1/1 Au–Ga–*R* AC (solid triangles), which has turned out to be difficult to be removed completely, such as by slight variation in the nominal composition and by change in the quenching conditions. The inset displays the selected-area electron diffraction patterns of *i* Au_65_Ga_20_Gd_15_ with incidence along the 2-fold, 3-fold, and 5-fold rotational-symmetry axes; the images clearly exhibit icosahedral symmetry features unique to *i* QCs. We note here that the τ scaling property observed in the 2-fold pattern indicates that the obtained *i* QC is a primitive *i* QC, identical to the prototype *i* Cd_5.7_Yb. Thus, we successfully obtained new *i* QCs with *e*/*a* = 1.70, which can be regarded as candidates of strong ferromagnetic *i* QCs based on the magnetic phase diagram recently obtained for Au–Al–Gd 1/1 ACs^22^ (see Figure 5).

### Observation of Ferromagnetic Transitions in *i* Au_65_Ga_20_Gd_15_ and *i* Au_65_Ga_20_Tb_15_

**Figure 3:**
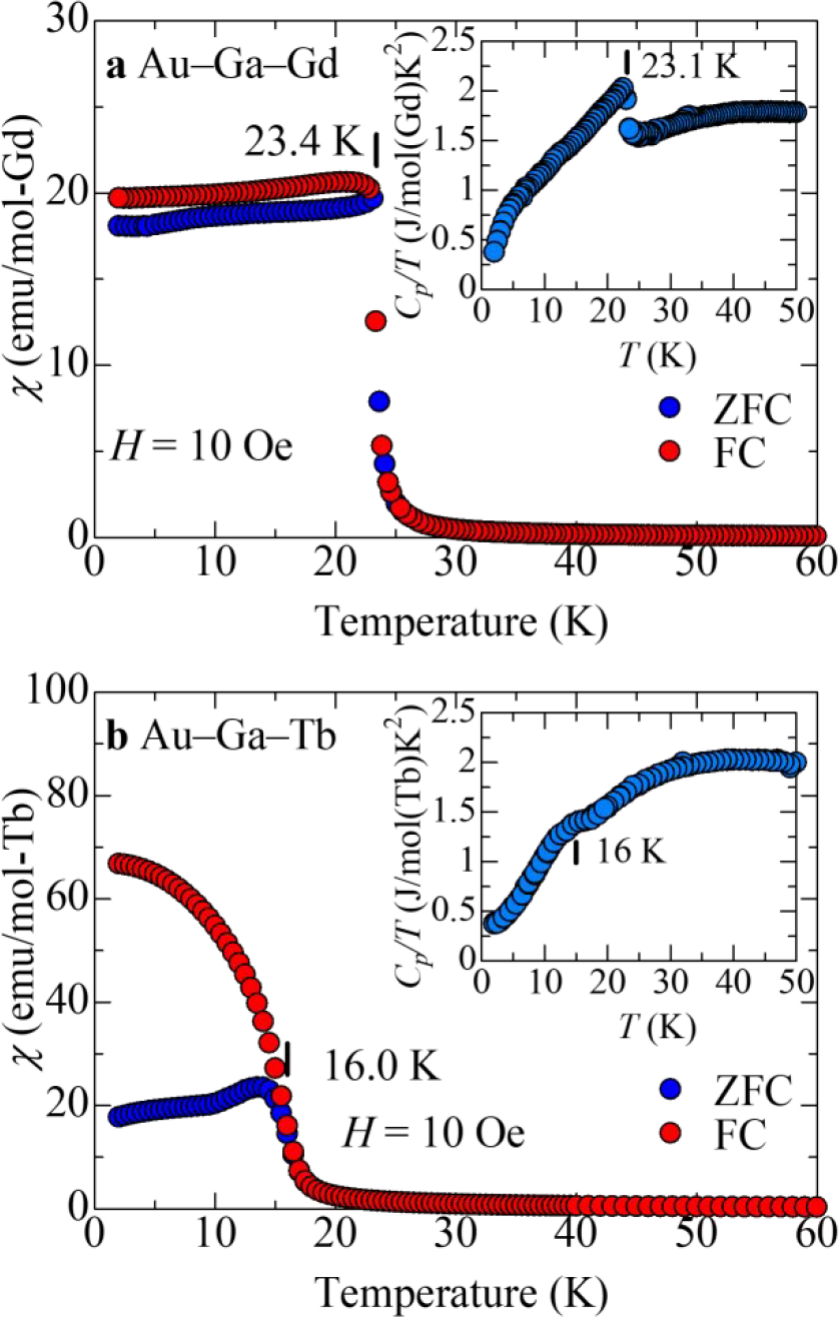
Temperature dependences of the FC and ZFC magnetic susceptibilities, χ = *M*/*H*, for (a) the Au_65_Ga_20_Gd_15_ and (b) Au_65_Ga_20_Tb_15_*i* QCs. FC and ZFC magnetic susceptibilities measured under 10 Oe are shown in the temperature range of 2–60 K. The insets show temperature dependences of specific heat, *C*_*p*_. In both insets, the Curie temperature *T*_C_ was estimated from the peak position of the d*C*_*p*_/d*T* curve.

Figures 3a and 3b show the magnetic susceptibility χ = *M*/*H* as a function of the temperature below 60 K for Au_65_Ga_20_*R*_15_ (*R* = Gd, Tb) *i* QCs, respectively, together with specific heat *C*_*p*_ in the temperature range of 2–50 K (insets).

The magnetic susceptibility clearly obeys the Curie–Weiss law χ = *N*_A_μ_eff_^2^/3*k*_B_(*T* – Θ) for both *i* QCs, where *N*_A_ denotes the Avogadro number, *k*_B_ the Boltzmann constant, and Θ the Weiss temperature (see Figure S1 of the Supporting Information). The effective magnetic moments μ_eff_ obtained from the fitting are 7.90 μ_B_ for *i* Au_65_Ga_20_Gd_15_ and 9.64 μ_B_ for *i* Au_65_Ga_20_Tb_15_, which are in good agreement with the theoretical values of *R*^3+^ (R = Gd, Tb) free ions, 7.94 μ_B_ and 9.72 μ_B_, respectively. Thus, the magnetic moments are strongly localized on the *R*^3+^ ions, as in the cases of the other *R*-containing *i* QCs. A distinct difference from the previously reported *R*-containing *i* QCs lies in the sign of Θ as a consequence of tuning the *e*/*a* ratio to 1.70, at which the largest Θ value was obtained for 1/1 Au–Al–Gd AC; the Θ values are 27.9 K for *i* Au_65_Ga_20_Gd_15_ and 12.9 K for *i* Au_65_Ga_20_Tb_15_, which clearly demonstrate that the interspin interactions are expectedly strongly ferromagnetic for these *i* QCs; this result is in contrast with the negative Θ values exclusively observed for all the other *i* QCs reported to date (see Figure 5).

From Figures 3a and 3b, we note that χ increases sharply at 23.4 and 16.0 K for the Au_65_Ga_20_*R*_15_ (*R* = Gd, Tb) *i* QCs, respectively, suggesting the occurrence of a ferromagnetic transition for both *i* QCs. For *i* Au_65_Ga_20_Tb_15_, a deviation of χ between the FC and ZFC susceptibilities is observed below 16.0 K; this behavior is similar to those observed for Tb-containing ferromagnetic ACs^21,23^ and is attributed to pinning of magnetic domain walls during the FC process. The *M*–*H* curves for Au_65_Ga_20_*R*_15_ (*R* = Gd, Tb) *i* QCs under fields up to 7 T at 2 K are provided in Figure S2. For *i* Au_65_Ga_20_Gd_15_, *M* quickly saturates to ∼7 μ_B_/Gd^3+^, nearly the full moment of a free Gd^3+^ ion (7 μ_B_/Gd^3+^), at a low field of 100 Oe, clearly showing that all the Gd^3+^ spins in the sample participate in the ferromagnetism and, hence, the majority QC phase is ferromagnetic. On the other hand, for *i* Au_65_Ga_20_Tb_15_, the *M* magnitude is suppressed to ∼6 μ_B_/Tb^3+^ at 7 T, about two-thirds of the full moment of a Tb^3+^ ion (9 μ_B_/Tb^3+^). This behavior including the *M* magnitude is, however, closely consistent with those of Tb-containing ferromagnetic ACs,^21−23^ which was attributed to the existence of the strong uniaxial anisotropy for the Tb^3+^ spins that have nonzero orbital angular momentum, i.e., *L* = 3, in sharp contrast with the Gd^3+^ spins with *L* = 0. The *M* suppression is ascribed to the formation of noncoplanar spin configuration due to this strong uniaxial anisotropy of Tb^3+^ spins in Au–Si–Tb 1/1 AC.^24^ The insets of Figures 3a and 3b show the variation in specific heat *C*_*p*_ of the Au_65_Ga_20_*R*_15_ (*R* = Gd, Tb) *i* QCs, respectively. For *i* Au_65_Ga_20_Gd_15_, *C*_*p*_ clearly displays a λ-shaped anomaly at 23.1 K, which well corresponds to the sharp increase in χ at 23.4 K; this result validates the magnetic transition occurrence, i.e., ferromagnetic transition, at *T*_C_ = 23 K. In contrast, for *i* Au_65_Ga_20_Tb_15_, *C*_*p*_ exhibits a broad anomaly around 16 K, close to the temperature corresponding to the sharp rise in χ. The reason for the broad anomaly is likely to be due to gradual development of the ferromagnetic order for *i* Au_65_Ga_20_Tb_15_. Its origin is not clear at present but might be related to the fact that *i* Au_65_Ga_20_Tb_15_ is located in the vicinity of the FM/AFM phase boundary as discussed later, where the existence of two competing phases may suppress sharp magnetic transition. On the other hand, *M*–*H* loops of *i* Au_65_Ga_20_Tb_15_ in Figure S3 clearly show hysteresis behavior below 16 K, a characteristic feature of ferromagnets; the remanence magnetization and the coercivity decrease with increasing temperature toward *T*_C_, which is consistent with the development of spontaneous magnetization below 16 K for *i* Au_65_Ga_20_Tb_15_. Moreover, the frequency dependence of *T*_c_ for *i* Au_65_Ga_20_Tb_15_ is found to be much smaller than that of *T*_f_ for the typical spin-glass *i* Zn–Mg–Tb,^25^ i.e., by 1 order of magnitude (see Figure S4), which rules out the possibility of spin-glass-like freezing for *i* Au_65_Ga_20_Tb_15_. Hence, we are consistently led to conclusion that both *i* Au_65_Ga_20_Gd_15_ and *i* Au_65_Ga_20_Tb_15_ exhibit a ferromagnetic transition at *T*_C_ = 23 and 16 K, respectively.

### Neutron Diffraction Experiments on *i* Au_65_Ga_20_Tb_15_ and *i* Au_65_Ga_20_Gd_15_

**Figure 4:**
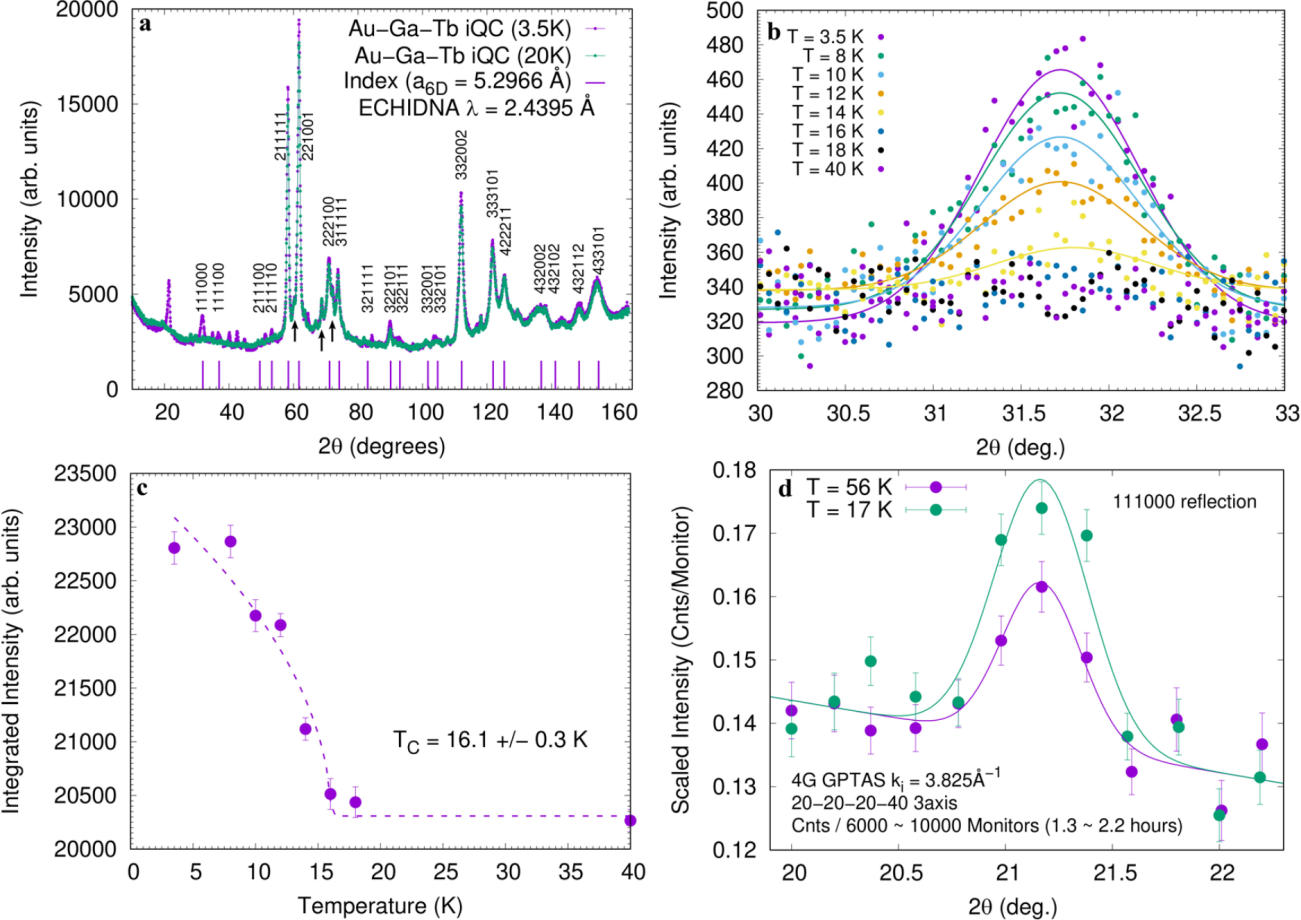
(a–c) Powder neutron diffraction patterns of the Au_65_Ga_20_Tb_15_*i* QC and (d) the Au_65_Ga_20_Gd_15_*i* QC. In (a), neutron diffraction patterns measured at the base temperature (3.5 K) and the paramagnetic temperature (20 K), which are below and above *T*_C_ = 16 K inferred from the bulk measurements, are shown, together with the nuclear peak positions and their 6D indices for the *i* QC calculated with the 6D lattice constant *a*_6D_ = 5.2966 Å. The vertical arrows indicate nuclear Bragg reflections from the impurity Au–Ga–Tb 1/1 approximant phase. The low-2θ region, which contains the strongest magnetic peak from the *i* QC, is magnified in (b). The magnetic 111000 peak is clearly observed at 2θ = 31.8° below *T*_C_ and disappears above *T*_C_, evidencing the formation of long-range magnetic order in the Au_65_Ga_20_Tb_15_*i* QC. The temperature dependence of the integrated intensity of the 111000 reflection is plotted in (c), in which a fit to an empirical function [*I* ∝ (1 – *T*/*T*_C_)^2β^] gives an estimate of the transition temperature as *T*_C_ = 16.1(3) K. In (d), the peak profile around the 111000 reflection from the *i* Au_65_Ga_20_Gd_15_ QC is shown for the two temperatures *T* = 17 and 56 K, i.e., below and above *T*_C_ = 23 K. The lines are a guide to the eye.

To gain further insight into the ferromagnetic transitions of *i* Au_65_Ga_20_Tb_15_ and *i* Au_65_Ga_20_Gd_15_, we additionally performed neutron diffraction experiments on both *i* QCs. Figure 4a shows powder neutron diffraction patterns of *i* Au_65_Ga_20_Tb_15_ measured at *T* = 3.5 and 20 K, i.e., below and above *T*_C_ = 16 K obtained from the bulk magnetization measurements.

Diffraction patterns measured at various temperatures are provided in the Supporting Information. We clearly observe magnetic Bragg reflections at the base temperature of 3.5 K. Some magnetic reflections newly appear at the base temperature, while many others are observed as an enhancement of the nuclear reflections (211111, 221001, and 332002, to note a few), confirming the ferromagnetic nature of the magnetic order. We note that several magnetic peaks at low angles are due to the contaminating 1/1 Au–Ga–Tb AC, the inclusion of which is detected in the XRD pattern (as denoted by the triangles in Figure 2) as well as in the neutron diffraction pattern (as denoted by the arrows in Figure 4a). Details of the appearance of the magnetic Bragg peaks from the 1/1 AC phase are given in Figure S6, where the powder neutron diffraction pattern of the Au–Ga–Tb 1/1 AC of the same nominal composition prepared by annealing at 923 K for 50 h is also provided. Here, it is noted that the magnetic reflections from the 1/1 Au–Ga–Tb AC can be consistently indexed with the cubic commensurate indices (see Figure S5). In addition, because of the loss of the bcc symmetry by the antiferromagnetic order, similar to the whirling magnetic order discovered in the 1/1 Au–Al–Tb AC,^26^ the magnetic reflections from the 1/1 Au–Ga–Tb AC appear separately from its nuclear reflections. Concerning this seemingly unexpected antiferromagnetic order for the Tb-bearing AC, we note that the antiferromagnetic/ferromagnetic phase boundary of Tb-bearing ACs has been found to be shifted significantly toward higher *e*/*a* ratios compared to Gd-bearing ACs, and *e*/*a* = 1.70 is very close to the phase boundary for Tb-bearing ACs.^27^ Thus, the occurrence of the antiferromagnetic order in the present 1/1 Au–Ga–Tb AC with *e*/*a* = 1.70 is not unexpected. For the difference in the magnetic order between the *i* QC and the 1/1 AC at the same *e*/*a* ratio, we note that the RKKY interaction depends on both the Fermi wavevector *k*_F_ and the spatial distribution of spins. Because the *e*/*a* ratio is related only to the Fermi wavevector *k*_F_, it is reasonable to consider that the different magnetic orders of the *i* QC and the 1/1 AC at the same *e*/*a* ratio are due to the difference in the spin arrangement between them. Not only the short-range but also the medium-range spin arrangement should play a role in the magnetism since the RKKY interaction is a rather long-range magnetic interaction slowly decaying with *r*^–3^. As for the difference in the spin arrangement between the *i* QC and the 1/1 AC, the following difference does exist: If we denote the spins on the second icosahedron shell as A and the spins inside the acute rhombohedron as B, the interspin distances in the *i* QC are aligned in the order of B–B, A–B, A–A, ..., from the nearest. We note that the nearest B–B and A–B pairs do not exist in the 1/1 AC, and therefore, not only medium-range but also short-range spin arrangement is considerably different between the *i* QC and the 1/1 AC.

In the *i* Au_65_Ga_20_Tb_15_ QC shown in Figure 4a, we clearly observe reflections that cannot be assigned to those of the 1/1 AC and that are located exactly at the positions of the nuclear Bragg reflections of the *i* QC, such as the 111000 and 111100 reflections (as newly appearing magnetic reflections) as well as 211111, 221001, and 332002 reflections (as reflections appearing on the nuclear reflection positions). Figure 4b magnifies the low-2θ region between 30° and 33°, wherein we observe the development of the strongest 111000 magnetic Bragg reflection with decreasing temperature below *T*_C_ = 16 K, which indicates ferromagnetic order formation in *i* Au_65_Ga_20_Tb_15_. Figure 4c shows the temperature evolution of the 111000 magnetic Bragg reflection intensity, wherein *T*_C_ is estimated as *T*_C_ = 16.1(3) K, which is in excellent agreement with the Curie temperature of *T*_C_ = 16 K observed in the bulk magnetic measurements.

In striking contrast to *i* Au_65_Ga_20_Tb_15_ which has relatively weak neutron absorption, neutron diffraction on *i* Au_65_Ga_20_Gd_15_ is one of the most difficult experiments due to the extraordinary absorption of the natural Gd atom. Nonetheless, we have succeeded in observing one magnetic Bragg peak in the ordered phase of *i* Au_65_Ga_20_Gd_15_ by employing a special thin-layer geometry for highly absorbing materials.^28^Figure 4d shows the neutron powder reflection profiles collected at the two temperatures *T* = 17 and 56 K, i.e., below and above *T*_C_ = 23 K, at the 111000 reflection position. It can be clearly seen that the reflection intensity increases below *T*_C_, where the ferromagnetic long-range order was observed in the bulk susceptibility and specific heat measurements. Although we could observe a single peak only, since it appears below the macroscopic *T*_c_, and also appears at the position which exactly matches to the 6D 111000 index, this observation is a solid evidence of the formation of the ferromagnetic order in *i* Au_65_Ga_20_Gd_15_. The temperature dependence of the 111000 peak intensity, as well as entire powder diffraction profiles with lower statistics, is given in Figure S7.

Here, we note that *this is the first direct microscopic observation of long-range magnetic order in QCs via neutron diffraction experiments*. The next epoch-making issue is to determine the complex magnetic structure of Au–Ga–Tb and Au–Ga–Gd *i* QCs. This requires the development of analysis methods and algorithms for the magnetic structure determination of ferromagnetic *i* QCs via higher-dimensional crystallography, and thus is left for the future study. Here, we just note that the magnetic structure in the Au–Ga–Tb *i* QC may not be simple collinear ferromagnetic, but may be rather noncoplanar ferrimagnetic, as suggested by the noncoplanar ferrimagnetic order recently found in the related Au–Si–Tb 1/1 AC.^24^

## Discussion

**Figure 5:**
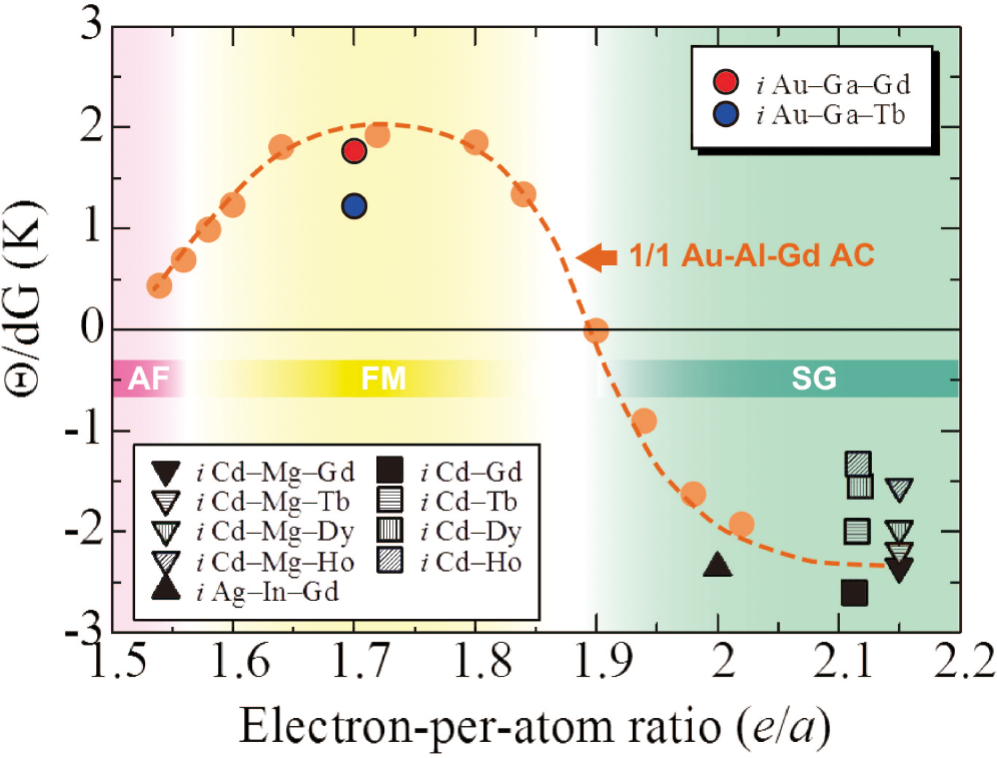
Normalized Weiss temperature, Θ/d*G*, and the magnetic ground state as a function of the *e*/*a* ratio. The Θ/d*G* values are plotted over a wide *e*/*a* range between 1.5 and 2.2 for all the *R*-containing Tsai-type *i* QCs reported to date, together with the Θ/d*G* values reported for the Au–Al–Gd 1/1 AC (orange circles) for comparison. The magnetic ground state of the Au–Al–Gd 1/1 AC is also shown. The Θ/d*G* values of the present Au–Ga–*R* (*R* = Gd, Tb) *i* QCs are large positive values, whereas those for the previously reported *i* QCs are large negative values without exception. This dependence of the Θ/d*G* value on the *e*/*a* ratio is in good agreement with the behavior observed in the Au–Al–Gd 1/1 AC.

First, we discuss the reason for the formation of the ferromagnetic QCs in the present Au_65_Ga_20_*R*_15_ (*R* = Gd, Tb) compounds. According to the theory underlying the Ruderman–Kittel–Kasuya–Yosida (RKKY) interaction,^28^ which is the major magnetic interaction between *R*^3+^ spins for *R*-containing QCs, the RKKY interaction magnitude scales with the de Gennes factor (dG) [(*g*_*J*_ – 1)^2^*J*(*J* + 1)], where *g*_*J*_ denotes the Landé *g*-factor and *J* the total angular momentum. Figure 5 shows the normalized Weiss temperature, Θ/d*G*, as a function of the *e*/*a* ratio over a wide *e*/*a* range from 1.5 to 2.2 for all the *R*-containing Tsai-type *i* QCs reported to date,^13,16,30^ together with the Θ/d*G* values for the Au–Al–Gd 1/1 ACs (orange circles) for comparison.^22^

The figure also shows the magnetic ground state regime of the Au–Al–Gd 1/1 AC in terms of the *e*/*a* ratio, wherein we note that the magnetic order changes from antiferromagnetic to ferromagnetic and then to spin glass (SG) with increasing *e*/*a* ratio. Here, we also note that this phase diagram has turned out to be applicable to Gd-bearing systems, and the antiferromagnetic/ferromagnetic phase boundary significantly shifts toward higher *e*/*a* ratios for Tb-bearing systems^27^ as mentioned above. From Figure 5, we clearly observe that the magnitude of the Θ/d*G* value and its sign are sensitively and systematically dependent on the *e*/*a* ratio for the AC. Consequently, it is clear that the negative Θ values exclusively observed for previously reported *i* QCs are attributed to their relatively large *e*/*a* values of 2.10–2.15, around which spin-glass-like behaviors are predominantly observed for both QCs and ACs. In contrast, the large positive Θ values for the present Au–Ga–*R i* QCs suggest that their *e*/*a* ratios (= 1.70) correspond to the middle of the ferromagnetic regime of the Au–Al–Gd 1/1 AC. Thus, our successful synthesis of ferromagnetic QCs justifies that the magnetic phase diagram obtained for ACs also holds for *i* QCs. This result means that *we have also obtained the long-sought conditions for realizing various magnetic orders including antiferromagnetic i QCs*. Moreover, our findings have shown that the Weiss temperature (or net magnetic interaction) is also tunable for *i* QCs via the tuning of the *e*/*a* ratio. Consequently, the quest for the first antiferromagnetic *i* QCs can now progress along this research line.

Ferromagnetic Au_65_Ga_20_*R*_15_ (*R* = Gd, Tb) *i* QCs are ordered solids with the highest possible symmetry, i.e., icosahedral symmetry; these QCs have not been synthesized previously. Hence, Au–Ga–*R i* QCs are the most isotropically ordered magnets among all materials discovered to date. For the icosahedral point groups (*I* and *I*_h_), there exist six 5-fold, ten 3-fold, and fifteen 2-fold axes, and accordingly, there can be 6, 10, and 15 easy axes, respectively, depending on the easy magnetization direction. The existence of such a large number of equivalent easy axes can result in significantly low-energy barriers between the neighboring easy axes (as in the case of cubic soft magnets), which can, in principle, lead to the more pronounced easy rotation of magnetic moments. From the technological viewpoint, isotropic materials should exhibit zero magnetocrystalline anisotropy energy, which results in low coercivity, low hysteresis loss, and high permeability. For icosahedral symmetry, the magnetic anisotropy energy is only associated with the higher-order terms (sixth-order and higher). In contrast, conventional crystals of the highest symmetry, i.e., cubic symmetry, where the fourth-order terms are nonzero, exhibit crystal anisotropy as the lower-order terms mostly contribute to the magnetic anisotropy. Thus, it would be a challenging issue to eliminate the sixth-order terms in the ferromagnetic *i* QC by tuning structural parameters via tweaking the *e*/*a* ratio and/or by isovalent substitution.

Finally, our successful synthesis of ferromagnetic *i* QCs shows that *e*/*a* tuning is effective in controlling the QC magnetism and that various exotic magnetic orders reflecting quasiperiodicity and/or high global/local symmetry can now be achieved by simply varying the *e*/*a* ratio. Moreover, our study opens pathways for the fundamental and technological exploration of the intrinsic nature of magnetic *i* QCs across various disciplines.

## Supporting Information Available

More details of the magnetization measurements and powder neutron diffraction experiments (PDF)
