# Supplementary material for: Experimental Observation of Long-Range Magnetic Order in Icosahedral Quasicrystals
Source: J Am Chem Soc. 2021 Nov 17;143(47):19938–44. doi: 10.1021/jacs.1c09954 (PMC8640986; doi:10.1021/jacs.1c09954)
Supplement: Supplementary file 1 — ja1c09954_si_001.pdf [file ja1c09954_si_001.pdf]

Supporting Information for "Experimental observation of long-range magnetic order in icosahedral quasicrystals"

Ryuji Tamura[1]*, Asuka Ishikawa[2], Shintaro Suzuki[1], Akihiro Kotajima[1], Yujiro Tanaka[1], Takehito Seki[3], Naoya Shibata[3], Tsunetomo Yamada[4], Takenori Fujii[5], Chin-Wei Wang[6], Maxim Avdeev[7,8] Kazuhiro Nawa[9], Daisuke Okuyama[9] and Taku J. Sato[9]*

[1]Department of Materials Science and Technology, Tokyo University of Science, Katsushika, Tokyo 125-8585, Japan,

[2]Research Institute for Science and Technology, Tokyo University of Science, Katsushika, Tokyo 125-8585, Japan,

[3]Institute of Engineering Innovation, School of Engineering, The University of Tokyo, Bunkyo, Tokyo 113-8656, Japan,

[4]Department of Applied Physics, Tokyo University of Science, Katsushika, Tokyo 125-8585, Japan,

[5]Cryogenic Research Center, The University of Tokyo, Bunkyo, Tokyo 113-0032, Japan,

[6]National Synchrotron Radiation Research Center, Hsinchu 30076, Taiwan,

[7]Australian Nuclear Science and Technology Organisation, New Illawarra Road, Lucas Heights, NSW 2234, Australia,

[8]School of Chemistry, The University of Sydney, Sydney, NSW 2006, Australia,

[9]Institute of Multidisciplinary Research for Advanced Materials, Tohoku University, 2-1-1 Katahira, Aoba, Sendai 980-8577, Japan

1. Experimental

1.1 Sample preparation and macroscopic measurements. Ternary $(Au,Ga)_{85}R_{15}$ (R = Gd, Tb) alloys with various Au/Ga ratios were prepared by arc-melting high-purity Au (99.99 wt%), Ga (99.9999 wt%), Gd (99.9 wt%), and Tb (99.9 wt%) raw elements. The nominal compositions were selected to ensure that electron-per-atom ratio (*e/a*) was close to 1.70, at which the highest Θ value was obtained for Au–Al–Gd 1/1 ACs[1]. The alloys were then rapidly quenched onto a Cu wheel rotating at 4000 rpm. The phase purity of the samples was examined via powder X-ray diffraction (Rigaku MiniFlex 600) with Cu *K*a radiation. Electron diffraction patterns were acquired by using a JEM-2010HC (JEOL Ltd.) microscope. The magnetic properties were measured by using a magnetic property measurement system (MPMS; Quantum Design) in the temperature range of 2–300 K under magnetic fields up to 7 T. Specific-heat measurements were performed by using a physical property measurement system (PPMS; Quantum Design) via the relaxation method between 2 and 50 K.

1.2 Neutron-diffraction experiments. Neutron-powder-diffraction experiments for the *i* $Au_{65}Ga_{20}Tb_{15}$ QC were performed by using the high-resolution powder diffractometer ECHIDNA installed at the OPAL reactor[2], Australian Nuclear Science and Technology

Organization. Neutrons with λ = 2.4395 Å were selected by using the Ge 331 reflections. The powder sample was loaded in a ϕ6 mm vanadium can and then set in a closed-cycle ^{4}He refrigerator with the base temperature of 3.5 K. Neutron-powder-diffraction experiment for the *i* $Au_{65}Ga_{20}Gd_{15}$ QC was performed by using the triple-axis spectrometer GPTAS installed at the JRR-3 reactor, Tokai, Japan. Neutrons with relatively short wavelength λ = 1.643 Å were selected by the pyrolytic graphite (PG) 002 reflections, and higher harmonic neutrons were eliminated by the PG filter. Only scattering in the elastic channel was collected using the PG 002 analyser to reduce the background. Collimations of 20'-20'-20'-40' were employed. The polycrystalline sample of *i* $Au_{65}Ga_{20}Gd_{15}$ was pulverized into fine powder, and was pasted on the single crystal Si plate using the CYTOP as a glue. The thickness of the Si plate was approximately 0.4 mm, whereas the powder sample thickness was approximately 0.05 mm. The Si plate was attached to the cold head of the closed-cycle ^{4}He refrigerator. The temperature of the Si plate was monitored by a directly attached thermometer, and is shown as the sample temperature in the manuscript. The diffraction was measured using the transmission geometry.

2. Magnetization measurements on $Au_{65}Ga_{20}R_{15}$ (*R* = Gd, Tb) *i* QCs

Figure S1 shows the inverse magnetic susceptibility $1/\chi$ = *H/M* as a function of the temperature from 2 to 300 K for $Au_{65}Ga_{20}R_{15}$ (*R* = Gd, Tb) *i* QCs. As can be seen from the linearity in both the $1/\chi - T$ curves, the magnetic susceptibility well obeys the Curie–Weiss law $\chi = N_A\mu_{eff}^2/3k_B(T-\Theta)$ for both *i* QCs, where N_A denotes the Avogadro number, k_B the Boltzmann constant, and Θ the Weiss temperature. The effective magnetic moments μ_{eff} obtained from the fitting are 7.90μ_B for *i* $Au_{65}Ga_{20}Gd_{15}$ and 9.64μ_B for *i* $Au_{65}Ga_{20}Tb_{15}$, which are in good agreement with the theoretical values of R^{3+} (*R* = Gd, Tb) free ions, 7.94μ_B and 9.72μ_B, respectively. The Θ values are 27.9 K for *i* $Au_{65}Ga_{20}Gd_{15}$ and 12.9 K for *i* $Au_{65}Ga_{20}Tb_{15}$, which shows that the inter-spin interactions are predominantly *ferromagnetic* for these *i* QCs, unlike all the other *i* QCs reported to date.

Figure S2 shows magnetic field dependences of the magnetization *M* measured at *T* = 2 K, for (a) the $Au_{65}Ga_{20}Gd_{15}$ and (b) $Au_{65}Ga_{20}Tb_{15}$ *i* QCs. For *i* $Au_{65}Ga_{20}Gd_{15}$, *M* quickly saturates to ~7μ_B/Gd^{3+}, nearly the full moment of a free Gd^{3+} ion (7μ_B/Gd^{3+}), at a low field of 100 Oe. On the other hand, for *i* $Au_{65}Ga_{20}Tb_{15}$, the *M* magnitude is suppressed to ~6μ_B/Tb^{3+} at 7 T, about two-thirds of the full moment of the Tb^{3+} ion (9μ_B/Tb^{3+}). Here, the quick magnetic saturation in *i* $Au_{65}Ga_{20}Gd_{15}$ is a characteristic

feature of ferromagnets with weak magnetic anisotropy. On the other hand, the M suppression in i $Au_{65}Ga_{20}Tb_{15}$ is due to a strong uniaxial anisotropy of Tb^{3+} spins (as described in the text).

Figure S3 shows M-H loops of i $Au_{65}Ga_{20}Tb_{15}$ measured at various temperatures below T_C = 16 K. A typical ferromagnetic hysteresis behaviour is observed; both the remanence magnetization and the coercivity decreases toward T_C, which is consistent with the development of spontaneous magnetization below T_C. Figure S4 shows the real part of the ac susceptibility of i $Au_{65}Ga_{20}Tb_{15}$ measured over a wide range of frequencies from 0.2 to 1000 Hz under the field of 1 Oe. The shift of the peak temperature is very small as described in the text, smaller than a typical Tb-bearing spin-glass 1/1 approximant by one order of magnitude.

3. Powder-neutron-diffraction experiments on *i* $Au_{65}Ga_{20}Tb_{15}$ and 1/1 $Au_{65}Ga_{20}Tb_{15}$.

Figure S3(a) shows the powder-neutron-diffraction patterns of i $Au_{65}Ga_{20}Tb_{15}$ measured at various temperatures in a range $3.5 < T < 40$ K, across the Curie temperature of T_C = 16 K observed in the bulk magnetic measurements. Below T_C = 16 K, new Bragg peaks, as well as enhancement of the reflection intensity at nuclear Bragg

positions, are clearly observed in a low-2θ region. A closer inspection of their temperature dependences reveals that there exist two sets of peaks with different transition temperatures. This is exemplified in Fig. S3(b), which shows a magnified diffraction pattern for 20° < 2θ < 33°. The lower-angle peak at 2θ = 21.4° disappears at 14 K, whereas the higher-angle peak at 2θ = 31.8° still retains some intensity at 14 K, indicating that the phase contributing to the lower-angle magnetic reflection has a slightly lower transition temperature below 14 K. Since the present *i* QC sample contains a small amount of 1/1 AC phase as probed by the powder x-ray diffraction (Fig. 2), we speculate that the secondary set of magnetic reflections with the lower transition temperature stems from the magnetic order of this contaminating 1/1 AC phase.

In order to verify this conjecture, we prepared a pure phase of the 1/1 Au-Ga-Tb AC with the same nominal composition as that of the *i* QC phase and performed neutron powder diffraction experiments on it. The single AC phase was obtained by annealing the sample, prepared by arc melting, at 923 K for 50 h. The neutron powder diffraction patterns of the 1/1 Au-Ga-Tb AC at the base temperature (3.5 K) and the paramagnetic temperature (20 K) are shown in Fig. S4. The diffraction pattern at 3.5 K clearly shows appearance of magnetic Bragg reflections, which can be indexed using

the antiferromagnetic modulation vector $\boldsymbol{q} = (1, 1, 1)$, similar to the case of the previously investigated 1/1 Au-Al-Tb AC[3]. Note that this magnetic modulation vector breaks bcc translational symmetry of the chemical lattice of the 1/1 AC, and therefore, the magnetic peaks appear in addition to the nuclear reflection positions.

By comparing the diffraction pattern of the 1/1 AC to that of the present *i* QC sample, one finds that the peaks appearing at $2\theta = 21.4°$, 34.8°, 40.0°, 42.4° and 44.7° are commonly observed in both the samples showing that these peaks *do* originate from the 1/1 AC as conjectured. On the other hand, the peaks at $2\theta = 31.8°$ and 36.9° were found to be observed only in the *i* QC sample. Since these reflections can be indexed with the 6D indices of primitive icosahedral lattice, such as 111000 and 111100 reflections, and their transition temperature (16.1(3) K) is exactly the same as that ($T_C = 16$ K) obtained from the bulk measurements, we have readily come to conclusion that the latter magnetic peaks are due to the *i* $Au_{65}Ga_{20}Tb_{15}$ and a long-range ferromagnetic order is established in *i* $Au_{65}Ga_{20}Tb_{15}$. The details of bulk magnetic properties and magnetic structure of the antiferromagnetic 1/1 AC phase will be reported elsewhere[2].

4. Powder-neutron-diffraction experiments on *i* $Au_{65}Ga_{20}Gd_{15}$.

Figure S7(a) shows the result of the powder-neutron-diffraction experiments on the *i* $Au_{65}Ga_{20}Gd_{15}$ QC. We note that due to the extraordinary absorption of natural Gd, only a small 2θ range around the 111000 reflection (denoted by the dotted box) was measured with sufficient statistics. The two peaks around θ ~ 38° and 40° are the two most intense reflections from the *i* QC phase, indicating that the diffraction signal is indeed from the *i* $Au_{65}Ga_{20}Gd_{15}$ QC. To compare, the powder X-ray diffraction pattern is also shown in the figure, with which the scattering angle was converted form the one for the Cu$K\alpha$ to the neutron λ = 1.643 Å. (Lattice shrinkage of 0.5% at low temperatures is also taken into account in the conversion of the X-ray diffraction pattern measured at the room temperature.) A good correspondence can be seen between the X-ray and neutron diffraction results. Figure S7(b) shows the temperature dependence of the peak intensity at the fixed 2θ ~ 21.2°. We note here that due to the insufficient statistics, we cannot reliably determine the magnetic transition temperature from the data; the line is only for the guide to the eye. Nevertheless, appreciable increase of the peak intensity can be seen below T_C = 23 K, indicating the development of magnetic contribution below T_C.

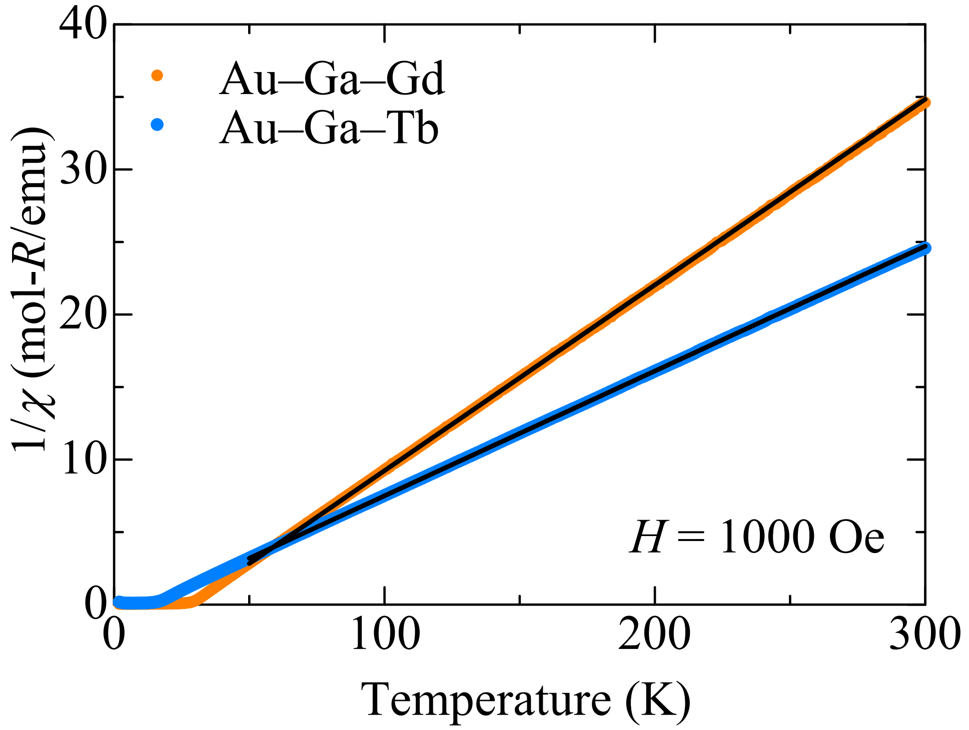

Figure S1. Temperature dependences of the inverse magnetic susceptibility $1/\chi = H/M$, for the $Au_{65}Ga_{20}R_{15}$ (R = Gd, Tb) *i* QCs. Magnetic susceptibilities measured under 1000 Oe are shown in the temperature range of 2–300 K. The solid black lines are fits to the Curie-Weiss law between 50–300 K.

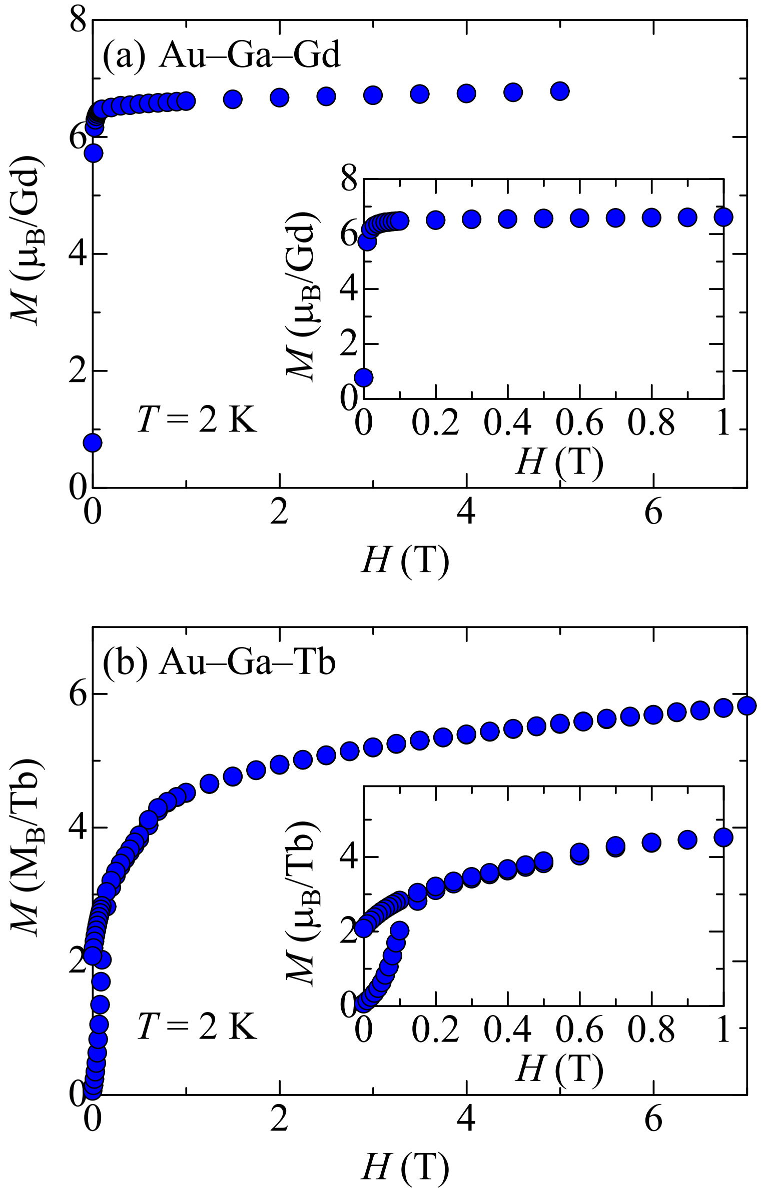

Figure S2. Magnetic field dependences of the magnetization *M*, for (a) the $Au_{65}Ga_{20}Gd_{15}$ and (b) $Au_{65}Ga_{20}Tb_{15}$ *i* QCs. Magnetizations of the $Au_{65}Ga_{20}Gd_{15}$ and $Au_{65}Ga_{20}Tb_{15}$ *i* QCs measured at 2 K are shown in the magnetic field range of 0 – 7 T. The insets show field dependences of the magnetization in the low field region.

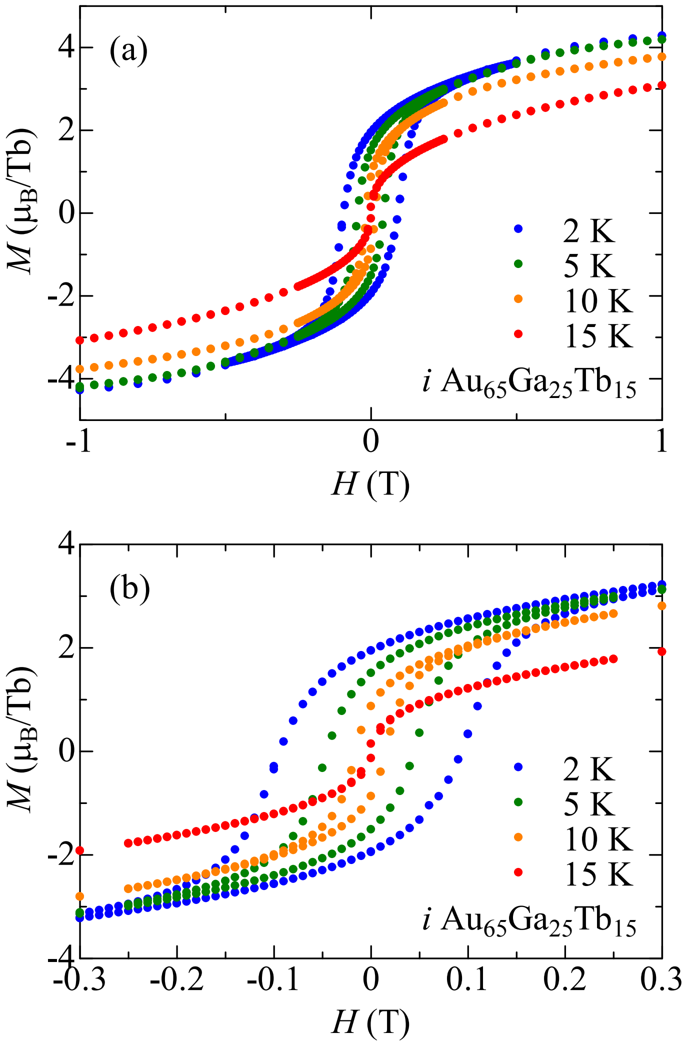

Figure S3. *M-H* loops measured below T_C = 16 K for $Au_{65}Ga_{20}Tb_{15}$ *i* QC. (a) Hysteresis loops of the $Au_{65}Ga_{20}Tb_{15}$ *i* QC measured at 2, 5, 10 and 15 K are shown in the magnetic field up to ± 1 T. (b) *M-H* loops magnified in the low field region between -0.3 and 0.3 T.

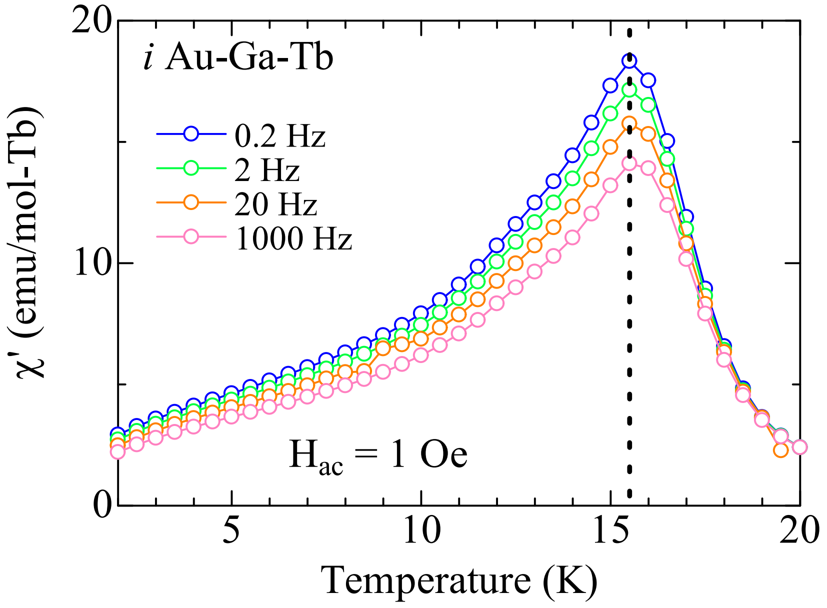

Figure S4. Real part of the ac susceptibility for $Au_{65}Ga_{20}Tb_{15}$ *i* QC. Real part of the ac susceptibility χ' measured at 0.2, 2, 20, and 1000 Hz under the field of 1 Oe for $Au_{65}Ga_{20}Tb_{15}$ *i* QC is shown.

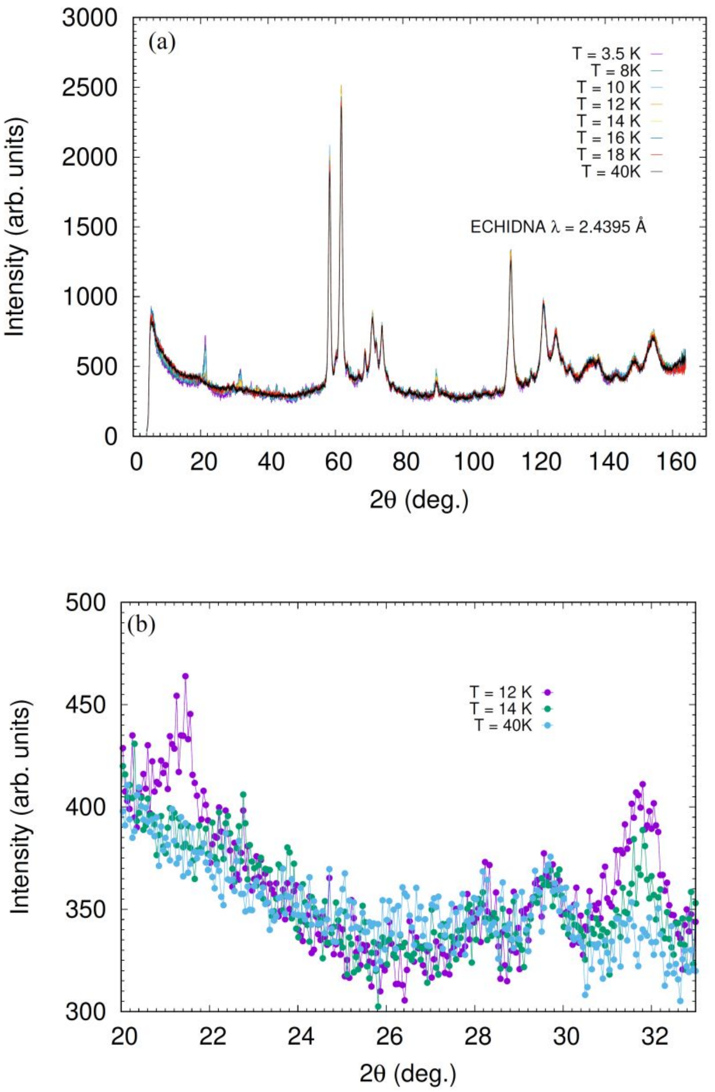

Figure S5. (a) Neutron-powder-diffraction patterns measured at various temperatures in 3.5 K < T < 40 K for the *i* Au-Ga-Tb QC sample. (b) Magnified pattern in the low-2θ region. Data at the selected temperatures across the anomaly temperature of T_C = 16 K observed in the bulk magnetic measurements are shown. It can be clearly seen that the peak at 21.4° disappears below T = 14 K, whereas that at 31.8° retains some intensity, implying that the peak at 21.4° is of a different origin.

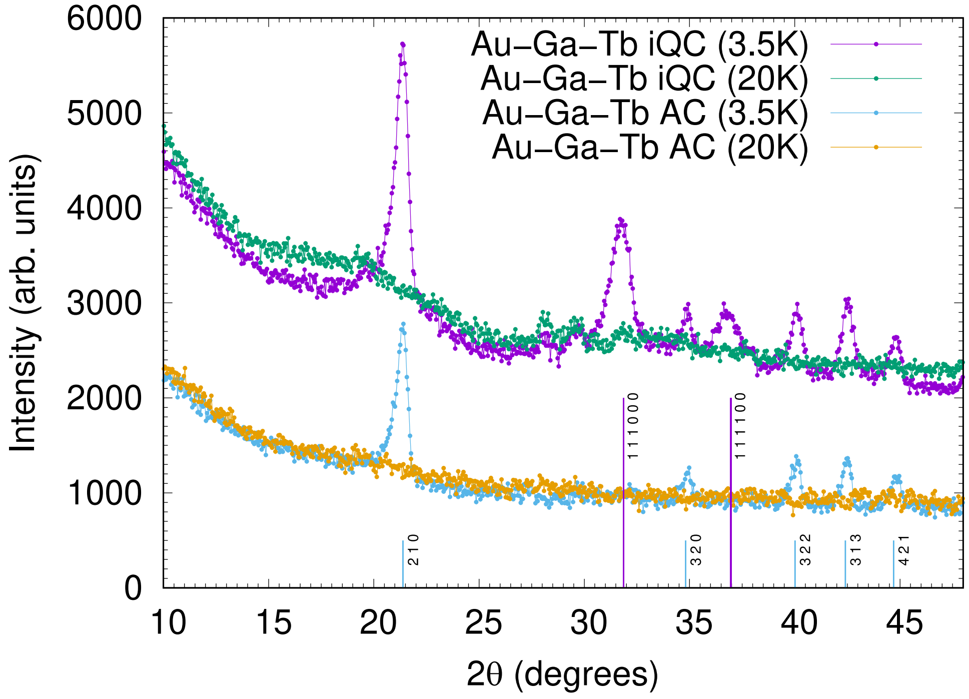

Figure S6. Neutron-powder-diffraction patters for the *i* QC and 1/1 AC samples at the base (3.5 K) and paramagnetic (20 K) temperatures. The light-blue and violet vertical lines represent the reflection positions of the antiferromagnetic AC phase and ferromagnetic *i* QC phase, respectively. Note that the peaks at 21.4°, 34.8°, 40.0°, 42.4° and 44.7° coincide with the 210, 320, 322, 313 and 421 magnetic peaks of the 1/1 AC, indicating that they are due to the antiferromagnetic 1/1 AC.

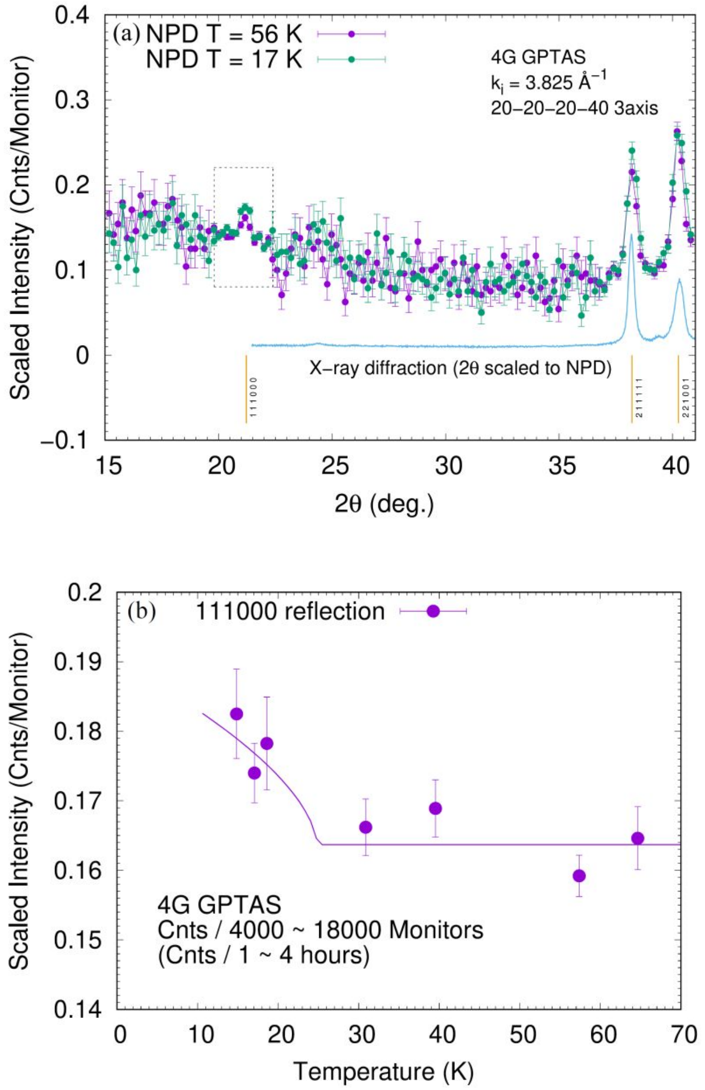

Figure S7. Neutron-powder-diffraction results for the *i* $Au_{65}Ga_{20}Gd_{15}$ QC. In **a**, the powder diffraction patterns at two temperatures $T = 56$ and 17 K are shown, together with the powder X-ray diffraction pattern scaled to the neutron wavelength $\lambda = 1.643$ Å. The vertical lines at the bottom represent the positions of the nuclear reflections with their 6D indices for *i* QC with the 6D lattice constant $a_{6D} = 5.315$ Å. In **b**, the temperature dependence of the peak intensity measured at $2\theta \sim 21.2°$ is shown.

References

1. Ishikawa, A.; Fujii T.; Takeuchi, T.; Yamada, T.; Matsushita, Y.; Tamura, R. Antiferromagnetic order is possible in ternary quasicrystal approximants. *Phys. Rev. B* **2018**, 98, 220403.
2. Avdeev, M.; Hester, J. R. ECHIDNA: a decade of high-resolution neutron powder diffraction at OPAL. *J. Appl. Crystallogr.* **2018**, 51, 1597-1604.
3. Sato, T. J.; Ishikawa, A.; Sakurai, A.; Hattori, M.; Avddeev, M.; Tamura, R. Whirling spin order in the quasicrystal approximant $Au_{72}Al_{14}Tb_{14}$. *Phys. Rev. B* **2019**, 100, 054417.
4. Nawa, K. *et al.* in preparation.
